# Prognostic significance of the angiopoietin-2 for early prediction of septic shock in severe sepsis patients

**DOI:** 10.2144/fsoa-2022-0077

**Published:** 2023-01-27

**Authors:** Luu Thi Thanh Duyen, Bui Van Manh, Tran Thi Phuong Thao, Le Van Khanh, Bui Ngoc Linh Trang, Ngo Truong Giang, Ha Van Quang, Nguyen Thanh Viet, Ngo Thu Hang, Can Van Mao, Nguyen Linh Toan, Hoang Van Tong

**Affiliations:** 1 Surgical Intensive Care Unit, Military Hospital 103, Vietnam Military Medical University, Hanoi, Vietnam; 2 Surgical Intensive Care Unit, Viet Tiep Hospital, Hai Phong, Vietnam; 3 Institute of Biomedicine & Pharmacy, Vietnam Military Medical University, Hanoi, Vietnam; 4 Department of Pathophysiology, Vietnam Military Medical University, Hanoi, Vietnam

**Keywords:** angiopoietin-1, angiopoietin-2, biomarker, sepsis, septic shock

## Abstract

**Aim:**

The current study investigated the plasma levels of angiopoietin-1/-2 and their association with clinical outcomes of sepsis.

**Methods:**

Angiopoietin-1 and -2 levels were quantified in plasma from 105 patients with severe sepsis by ELISA.

**Results:**

Angiopoietin-2 levels elevated according to the severity of sepsis progression. Angiopoietin-2 levels were correlated with mean arterial pressure and platelets counts, total bilirubin, creatinine, procalcitonin, lactate levels and SOFA score. Angiopoietin-2 levels accurately discriminated for sepsis with an AUC = 0.97 and septic shock from severe sepsis patients (AUC = 0.778).

**Conclusion:**

Plasma angiopoietin-2 levels may serve as an additional biomarker for severe sepsis and septic shock.

## Plain language summary

The study investigated the plasma levels of angiopoietin-1/-2 and their association with clinical outcomes of sepsis in plasma from 105 patients with severe sepsis by ELISA. The results showed that angiopoietin-2 levels elevated according to the severity of sepsis progression and were correlated with important clinical parameters such as mean arterial pressure and platelets counts, procalcitonin, lactate levels and SOFA score. Angiopoietin-2 levels accurately discriminated for sepsis and septic shock. Thus, plasma angiopoietin-2 levels may serve as an additional biomarker for severe sepsis and septic shock.

Septic shock is a serious disease with a high mortality rate, ranking first in the intensive care units (ICUs) [1]. The causes of sepsis are highly diverse, depending on the geographical area, hospitals, pathogenic profile and the rate of drug resistance [1]. Septic shock is the most severe form of bloodstream infection that begins with a systemic inflammatory response (SIRS) and consequently leads to multiple organ failure [2,3]. Septic shock is characterized by a systemic inflammatory response accompanied by fever, hypotension, cardiovascular dysfunction and impaired tissue perfusion as well as decreased oxygen delivery that leads to alterations in the intrinsic function of the microcirculation, which play an important role in the pathogenesis of multiple organ failure and subsequent mortality [4,5]. Septic shock at a late stage, when there are many complications, the treatment becomes more complicated and increases the mortality rate, and the risk of death can be up to 40–60% [6,7]. Early diagnosis and prognosis of septic shock mainly based on clinical evidence when blood culture results are not available is essential to decide on the right and timely treatment [2]. However, diagnosis and determination of severity in the early stages of sepsis are difficult because clinical symptoms are often nonspecific, especially in elderly patients. Thus, septic shock remains one of the leading causes of death for sepsis patients. The proportion of patients with septic shock, the rate of hospitalized patients and the mortality rate due to septic shock are increasing [7].

Among the methods of diagnosing sepsis and monitoring septic shock, blood culture is currently considered the gold standard for the detection of pathogenic infections. Common pathogens that cause sepsis include *Escherichia coli, Staphylococcus aureus, Pseudomonas aeruginosa, Enterococcus faecalis, Streptococcus pneumoniae, Klebsiella pneumoniae*, and *Enterobacter* species [7,8]. However, approximately 10% of patients with sepsis are not identifying infectious pathogens. Therefore, a number of serum biomarkers have been proposed to predict septic shock in sepsis patients such as C-reactive protein (CRP), cytokines (IL-1, IL-6, IL-10 and TNF-α), chemokines (IL-8, MCP-1, G-CSF) and procalcitonin [3,9]. Among them, procalcitonin has been used to differentiate between bacterial and viral infections [10]. However, these biomarkers cannot help diagnose, early, and prognosticate septic shock and also differentiate it from some other diseases such as systemic inflammatory response syndrome (SIRS) [3]. Therefore, new biomarkers that are important and specific for septic shock are necessary for the diagnosis and prognosis of the development of septic shock in sepsis patients as well as to differentiate between SIRS and septic shock.

Angiogenesis is related to many developmental processes such as germination, endothelial cell migration, proliferation and destabilization, and vascular formation. Angiopoietins are a family of endogenous vascular growth factors essential for embryonic and postnatal angiogenesis and stabilization [11,12]. Angiopoietins involves in the binding and disassembling of the endothelial cells of blood vessels [13]. Thus, angiopoietin signalling is involved in the control of capillary permeability, vasodilation and vasoconstriction by interacting with the smooth muscle cells surrounding the vessels. Two members of the angiopoietin family that have been studied extensively in humans are angiopoietin-1 and angiopoietin-2. Both angiopoietins bind to the angiopoietin receptor (TIE-2) on endothelial cells, angiopoietin-1 is a TIE-2 agonist, while angiopoietin-2 is a contextual antagonist of TIE-2. In vasculature, angiopoietin-1 protects against vascular leakage, while angiopoietin-2 promotes increased vascular permeability [12,14,15]. Angiopoietin-1 is important for vascular maturation, adhesion, migration and survival while angiopoietin-2 promotes cell death and vascular disruption [16]. However, when it is combined with vascular endothelial growth factor (VEGF), it can promote neovascular growth [11].

The plasma levels of angiopoietins have been shown to be associated with several diseases and disorders such as acute sepsis-induced lung injury and congestive heart failure [17–19]. In bloodstream infection, the angiopoietin-2/TIE-2 receptor complex may be an important target in patients with septic shock and multiple organ dysfunction syndromes (MODS) [20]. Therefore, angiopoietin-2 may serve as an important biomarker of endothelial injury. However, the prognostic significance of the angiopoietins for early prediction of septic shock in sepsis patients needs to be validated. The current study aims to investigate the plasma levels of angiopoietin-1 and angiopoietin-2 in patients with severe sepsis and septic shock and their association with the clinical outcome of septic shock in Vietnamese patients. The results could provide more evidence of diagnostic and prognostic values of these biomarkers for the management of patients with severe sepsis and septic shock.

## Materials & methods

### Study subjects

This was a prospective observational study performed at a single center to evaluate the plasma levels of angiopoietin-1/-2 and their association with clinical outcomes of sepsis. All patients with culture-confirmed sepsis admitted to the intensive care unit (ICU) of the Viet Tiep Hospital, Hai Phong, Vietnam from October 2018 to December 2020 were considered for this study. One hundred and five (n = 105) Vietnamese patients with severe sepsis were recruited for this study. The mean age was 66.16 ± 16.34 years; 44.76% of patients <65 years old and 57 (53.33%) were male. These patients were further classified into two different groups based on clinical symptoms, the severity of the disease and the presence of septic shock. The first group included 52 patients who were diagnosed with severe sepsis, and the second group included 53 severe sepsis patients who had septic shock. Sepsis, severe sepsis and septic shock patients were diagnosed and classified according to the clinical practice guidelines of the American College of Chest Physicians/Society of Critical Care Medicine (ACCP/SCCM) [21]. Accordingly, severe sepsis is characterized by the combination of organ dysfunction and infection, of which, organ dysfunction is determined by either acute change in SOFA score ≥2 at Intensive care units (in ICUs) or quick Sequential Organ Failure Assessment (SOFA) score ≥2 points (outside ICUs). Septic shock is characterized by all the symptoms of sepsis plus persistent hypotension requiring vasopressors (to maintain systolic blood pressure ≥65 mmHg) and blood lactate level ≥2 mmol/l (despite adequate fluid resuscitation) [21]. All patients were confirmed positive for blood culture test. We excluded patients with diseases that can affect angiopoietin levels such as dengue fever, cerebral stroke and acute renal failure [22–24]. After hospital admission, patients were treated with antibiotics and vasopressors for patients with septic shock and other supportive treatments. All patients were followed up, and biochemical and clinical parameters and blood samples were collected at three time points including at hospital admission (T0), 3 days (T1) and 7 days (T2) after hospital admission. All laboratory tests including blood counts (red blood cells, white blood cells, platelets), blood gas and laboratory tests for organ functions such as bilirubin, pro-calcitonin, albumin, creatinine, lactate and were determined by laboratory routine procedures.

As a control group, 95 Vietnamese healthy individuals of the same ethnicity living in the north of Vietnam were recruited at the Vietnam Military Medical University. These control individuals were confirmed negative for HBsAg, anti-HCV, and anti-HIV by routine serological procedures. These control individuals had no history of any febrile or other illnesses in the previous 3 months. In addition, control individuals also had no history of chronic diseases such as diabetes, hepatitis, heart disease or any infection, nor any alcohol and/or drug usage. Approximately 5 ml of venous blood were collected from all patients and healthy controls and the serum and/or plasma were separated from whole blood. Aliquots were transferred to a fresh polypropylene tube and were stored at -80°C until use.

### Collection of clinical & subclinical parameters

We recorded all clinical, biochemical and laboratory parameters of the study subject at three time points including at hospital admission (T0), 3 days (T1) and 7 days (T2) after hospital admission. The Glasgow Coma Scale (GCS) was calculated to assess the consciousness of patients based on eyes, verbal and motor responses. The Sequential Organ Failure Assessment (SOFA) score was calculated based on the laboratory and clinical tests of respiratory, coagulation, cardiovascular, nervous and kidney functions. The Acute Physiology and Chronic Health Evaluation (APACHE) II score was calculated based on body temperature, mean arterial pressure, heart rate, respiratory rate, oxygenation, arterial pH, sodium, potassium, creatinine, hematocrit, white blood cell count, Glasgow Coma Scale (GCS) score, age, chronic diseases and surgical status, with a total possible score between 0 and 71 [25].

### Quantification of the levels of Angiopoietin-1 & Angiopoietin-2

The plasma Angiopoietin-1 levels were measured in plasma samples from patient groups and healthy controls using the Human ProcartaPlex Mix&Match 4-plex (Thermo Fisher Scientific, Waltham, MA, USA; catalog no.: PPX-04-MXWCXE3) following manufacturer's instructions. The plasma Angiopoietin-2 levels were measured in plasma samples from patient groups and healthy controls using the Human Angiopoietin-2 ELISA Kit (Thermo Fisher Scientific; catalog no.: KHC1641) following the manufacturer's instructions.

### Statistical analysis

Demographic and clinical characteristics were presented as medians with range for continuous variables and number with percentage for categorical variables. Categorical data were compared using Chi-square or Fisher's exact tests and continuous data were compared using Student's *t*-test or Mann-Whitney U test, where appropriate. Mann Whitney U or Kruskal–Wallis tests were used to compare serum Angiopoietin-1 and Angiopoietin-2 levels between groups. Spearman's rank correlation coefficient was used to analyze the correlation between two studied variables. A receiver operating characteristic (ROC) curve and areas under the ROC curves were used to assess the prognostic significance of the variables. Furthermore, the optimal cutoff value for Angiopoietin-1 and Angiopoietin-2 levels to differentiate between server sepsis and septic shock. Data was organized by Microsoft office Excel-2010 and analyzed by SPSS v.22 software (SPSS Statistics, IBM, NY, USA). Two-sided p values less than 0.05 were considered statistically significant.

## Results

### Demographic, biochemical & clinical characteristics of the study subjects

The demographic, biochemical and clinical characteristics of the severe sepsis patients, and patients with septic shock at hospital admission and control individuals are summarized in Table 1. Although the age of the patient group was higher compared with controls, no statistical differences in age between severe sepsis patients (69.5 years) and patients with septic shock (66 years) were observed. Regarding gender, no significant difference in gender distribution among study groups was observed. Within the group of patients, the mean arterial pressure and platelet counts of patients with septic shock were significantly decreased compared with those with severe sepsis (p < 0.0001). In contrast, the levels of total bilirubin, blood creatinine, procalcitonin and lactate were significantly higher in patients with septic shock compared with those with severe sepsis (p < 0.05). In addition, the SOFA score of patients with septic shock was higher compared with those with severe (p = 0.018), whereas no difference in APACHE II score between these two groups of patients was observed.

**Table 1.** Characteristics of sepsis patients and controls at hospital admission.

Characteristics	Healthy controls (n = 95)	Patients at hospital admission (T0)			p^†^ value
		Severe sepsis (n = 52)	Septic shock (n = 53)	p^‡^ value	
Age (years)	40 [19–75]	69.5 [16–90]	66 [38–91]	NS	<0.0001
Gender (male/female)	44/51	27/25	30/23	NS	NS
Mean arterial pressure (mmHg)	NA	91.7 [50–123.3]	71.7 [49–118]		NA
Hematocrit (%)	138 [104–169]	31.3 [20–44.4]	33.1 [17.8–53.9]	NS	NS
White blood cells (g/l)	6.5 [3.6–12.5]	13.95 [4.5–61.1]	14.5 [0.4–64.3]	NS	NS
Platelets (g/l)	242 [150–385]	198 [25–547]	106 [2–516]	<0.0001	<0.0001
Total bilirubin (μmol/l)	9.2 [3.1–25.7]	16.3 [6.3–202.9]	29.2 [5–205.1]	0.02	<0.0001
Blood creatinine (μmol/l)	77 [52.2–146.99]	76.25 [41.6–772]	150.05 [53.9–692.1]	<0.0001	<0.0001
Albumin (g/l)	NA	28.5 [17.8–118.4]	27.9 [15–45.3]	NS	NA
Procalcitonin (ng/ml)	NA	9.35 [0.32–200]	51.5 [3.5–200]	<0.0001	NA
Lactate (mmol/l)	NA	1.36 [1–2]	5.55 [2–18]	<0.0001	NA
Glasgow coma scale	NA	2 [0–4]	1 [0–4]	NS	NA
SOFA score	NA	5 [1–10]	6 [1–15]	0.018	NA
APACHE II score	NA	16.5 [3–27]	15 [5–28]	NS	NA
Ang-1 (pg/ml)	91.6 [1.65–1405.9]	334.57 [32–6498.7]	165.9 [11.66–2459.5]	0.042	<0.0001
Ang-2 (pg/ml)	105.5 [21.9–337.2]	515.56 [76.2–1291.3]	1096.76 [44.2–1511.9]	<0.0001	<0.0001
Ang-1/Ang-2 ratio	0.8 [0.08–8.4]	0.53 [0.034–20.6]	0.14 [0.014–16.99]	0.001	<0.0001

† Comparison between patients and control individuals;

‡ Comparison between severe sepsis patients with septic shock patients.

Values given are medians and range.

APACHE II: Acute Physiology and Chronic Health Evaluation II; Ang: Angiopoietin; NA: Not applicable; NS: Not significant; SOFA: Sequential Organ Failure Assessment.

### Levels of Angiopoietin-1 & Angiopoietin-2 in patients with severe sepsis & septic shock

**Figure 1.**
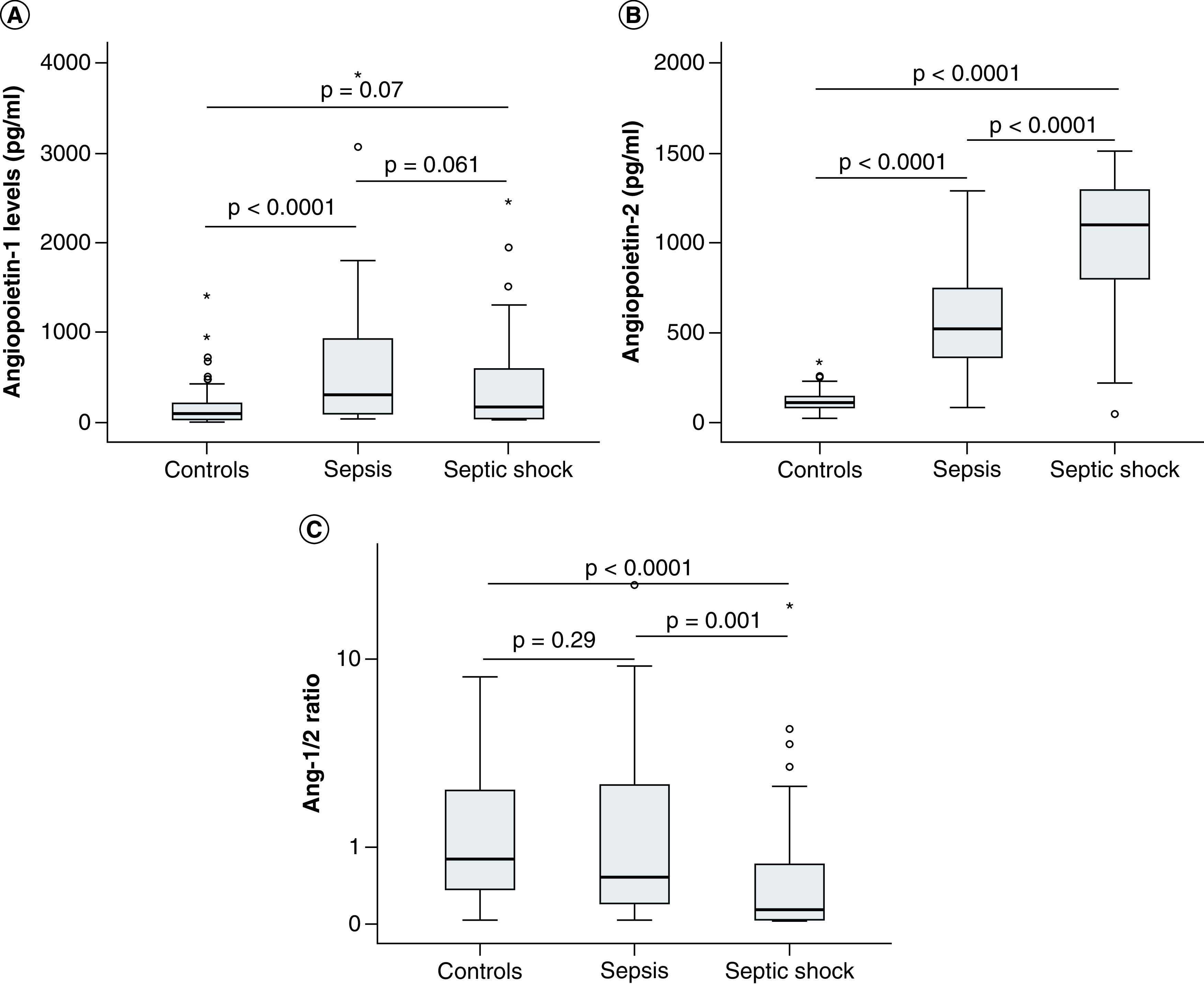
The levels of angiopoietin-1 and angiopoietin-2 in patients and control individuals. **(A)** The levels of angiopoietin-1 **(B)** and angiopoietin-2 were measured in the serum samples from sepsis patients, those with septic shock at hospital admission, as well as in control individuals. **(C)** The ratios of angiopoietin-1 to angiopoietin-2 levels (Ang-1/Ang-2 ratio) were calculated for each patient and control individual and were compared between groups. p values were calculated by using the Mann-Whitney U test.

The levels of both angiopoietin-1 and angiopoietin-2 were significantly increased in sepsis patients compared with control individuals at the time of hospital admission (T0; p = 0.0001). When separated patients into two subgroups including severe sepsis patients and those with septic shock, angiopoietin-1 levels were significantly increased in severe sepsis patients (334.57 pg/ml) compared with control individuals (91.6 pg/ml) (p < 0.0001) while there was no significant difference in angiopoietin-1 levels between patients with septic shock (165.9 pg/ml) and control group (p = 0.07). Angiopoietin-2 levels were significantly increased in both severe sepsis patients (515.56 pg/ml) and those with septic shock (1096.76 pg/ml) compared with control individuals (105.5 pg/ml) (p < 0.0001). When compared angiopoietin levels between the two subgroups of patients, angiopoietin-2 were significantly increased in patients with septic shock compared with severe sepsis patients (p < 0.0001), while no significant difference in angiopoietin-1 levels was observed (p = 0.061). In addition, we also calculated the ratios of angiopoietin-1 to angiopoietin-2 levels (Ang-1/2 ratios) and compared among three study groups. The results showed that the Ang-1/2 ratios were significantly lower in patients with septic shock (Ang-1/2 ratios = 0.14) compared with severe sepsis patients (Ang-1/2 ratios = 0.53) and control individuals (Ang-1/2 ratios = 0.8) (p = 0.001 and <0.0001, respectively) (Table 1 & Figure 1).

**Figure 2.**
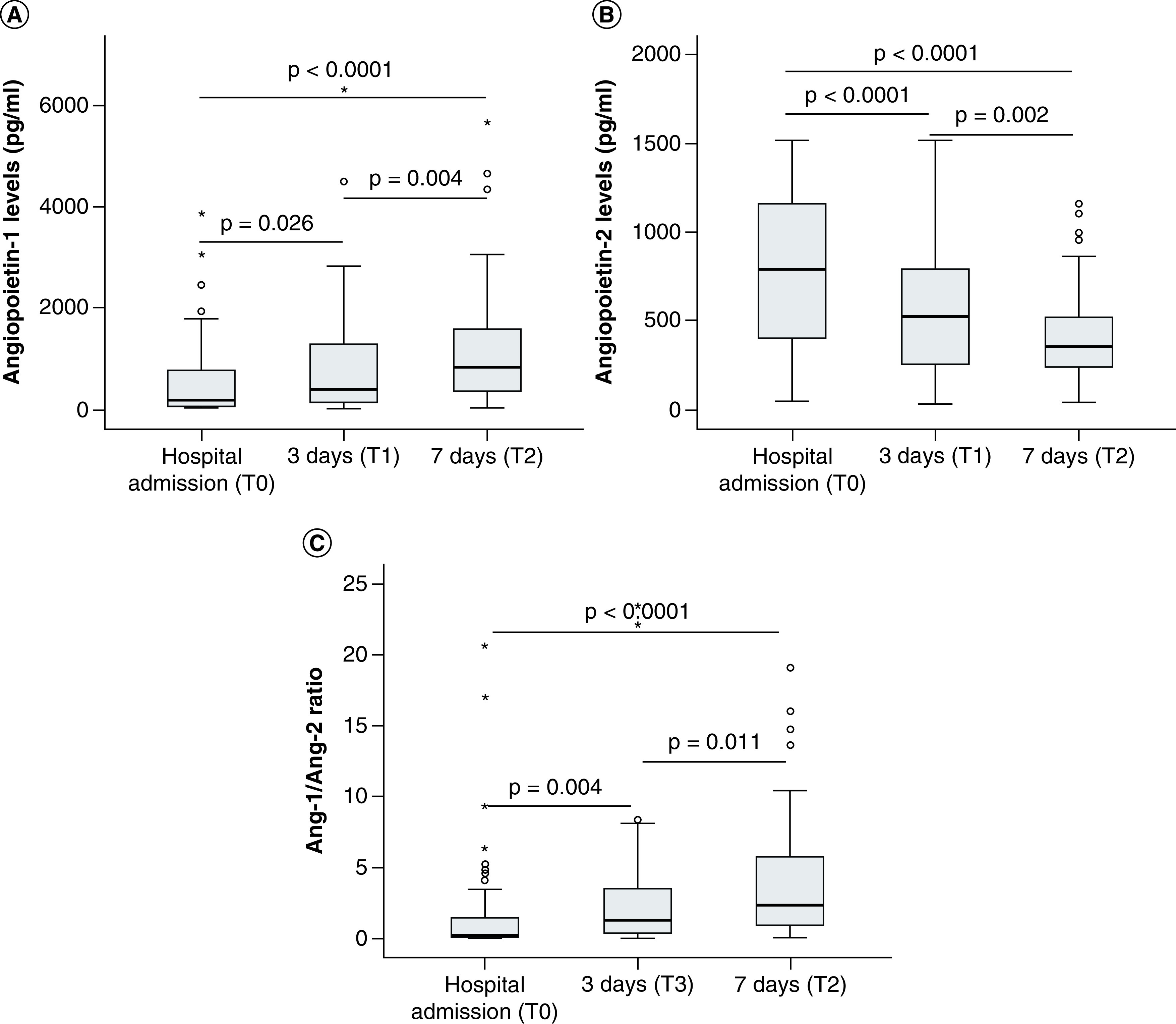
The levels of angiopoietin-1 and angiopoietin-2 in patients at different time points. **(A)** The levels of angiopoietin-1 **(B)** and angiopoietin-2 were measured in the serum samples from sepsis patients, those with septic shock at different time points (hospital admission, 3 and 7 days after hospital admission). The ratios of angiopoietin-1 to angiopoietin-2 levels (Ang-1/Ang-2 ratio) were calculated for each patient and control individual and were compared between groups **(C).** p values were calculated by using the Mann-Whitney U test.

We also measured the angiopoietin levels at three (T1) and seven (T2) days after hospital admission and compared with those at the timepoint of hospital admission (T0). The results showed that angiopoietin-1 levels were significantly increased (p = 0.026 and 0.004, respectively). In contrast, angiopoietin-2 levels were significantly decreased after three (T1) and seven (T2) days of hospital admission compared with those at the timepoint of hospital admission (T0) (p < 0.0001 and 0.002, respectively). Also, the Ang-1/2 ratios were significantly increased after three (T1) and seven (T2) days of hospital admission compared with those at the timepoint of hospital admission (T0) (p < 0.0001 and 0.011, respectively) (Figure 2).

### Correlation of Angiopoietin-1 & Angiopoietin-2 levels with clinical parameters

We analyzed the correlation of the levels of angiopoietin-1, angiopoietin-2 and Ang-1/2 ratios with the clinical parameters of sepsis (Table 2 & Supplementary Data). At hospital admission (T0), we observed that the angiopoietin-1 levels were positively correlated with platelets counts (Spearman's rho = 0.29, p = 0.012) and negatively correlated with total bilirubin levels (Spearman's rho = -0.38, p = 0.0017) (Supplementary Data). Whereas, the angiopoietin-2 levels were negatively correlated with mean arterial pressure (Spearman's rho = -0.42, p < 0.0001) and platelets counts (Spearman's rho = -0.232, p = 0.047), and positively correlated with total bilirubin levels (Spearman's rho = 0.37, p = 0.0025), creatinine levels (Spearman's rho = 0.43, p = 0.00012), procalcitonin levels (Spearman's rho = 0.56, p < 0.0001), lactate levels (Spearman's rho = 0.47, p = 0.0004) and SOFA score (Spearman's rho = 0.41, p = 0.0002). We also observed the correlation of Ang-1/2 ratios with mean arterial pressure (Spearman's rho = 0.28, p = 0.012) and platelets counts (Spearman's rho = 0.36, p = 0.0014), total bilirubin levels (Spearman's rho = -0.49, p < 0.0001), procalcitonin levels (Spearman's rho = -0.29, p = 0.01) and lactate levels (Spearman's rho = -0.29, p = 0.037) (Supplementary Data). At the timepoint of 3 days after hospital admission (T2), we observed the correlation of both angiopoietin-1 and angiopoietin-2 levels with platelets counts, total bilirubin, creatinine, procalcitonin and lactate levels. At the timepoint of 7 days after hospital admission (T2), we observed the correlation of angiopoietin-2 levels with hematocrit, total bilirubin, creatinine, procalcitonin and albumin levels, while angiopoietin-2 levels were correlated with mean arterial pressure, platelets counts and total bilirubin levels (Table 2 & Supplementary Data).

**Table 2.** Correlation of Angiopoietin-2 levels with clinical parameters.

Clinical parameters	Correlation	Hospital admission	3 days after hospital admission	7 days after hospital admission
Mean arterial pressure (mmHg)	ρ (rho)	**-0.42**	**-0.3**	-0.15
	p value	**<0.0001**	**0.012**	0.52
White blood cells	ρ (rho)	0.08	0.19	0.06
	p value	0.51	0.18	0.7
Platelets (g/l)	ρ (rho)	**-0.232**	**-0.57**	-0.08
	p value	**0.047**	**<0.0001**	0.63
Hematocrit (%)	ρ (rho)	0.04	-0.02	**-0.37**
	p value	0.73	0.89	**0.023**
Total bilirubin (μmol/l)	ρ (rho)	**0.37**	**0.51**	**0.33**
	p value	**0.0025**	**0.0003**	**0.059**
Blood creatinine (μmol/l)	ρ (rho)	**0.43**	**0.33**	**0.45**
	p value	**0.00012**	**0.011**	**0.003**
Procalcitonin (ng/ml)	ρ (rho)	**0.56**	**0.42**	**0.39**
	p value	**<0.0001**	**0.0004**	**0.008**
Albumin (g/l)	ρ (rho)	-0.15	-0.18	**-0.36**
	p value	0.18	0.15	**0.014**
Lactate (mmol/l)	ρ (rho)	**0.47**	**0.46**	-0.17
	p value	**0.0004**	**0.0024**	0.54
SOFA score	ρ (rho)	**0.41**	-0.01	0.18
	p value	**0.0002**	0.93	0.2
APACHE II score	ρ (rho)	0.05	0.13	0.23
	p value	0.65	0.29	0.09

The correlations between the pair of studied parameters were calculated by using Spearman's rank correlation coefficient. Spearman's rho (ρ) and p values are presented.

Bold values are statistical significant.

### Prognostic significance of Angiopoietin-1 & Angiopoietin-2 levels for septic shock

**Figure 3.**
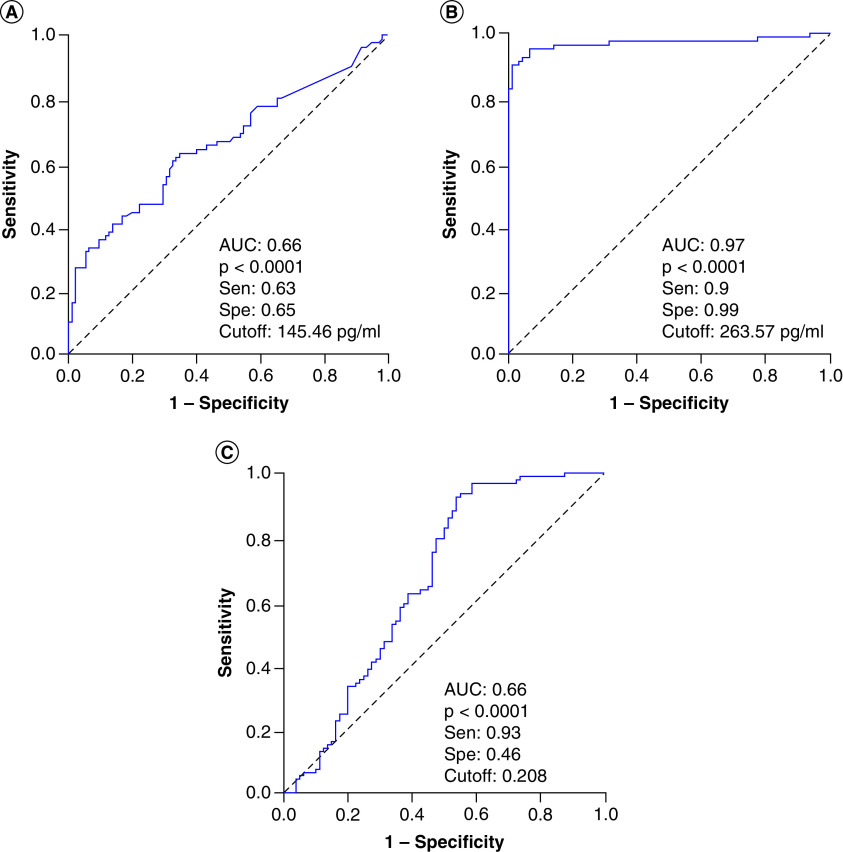
Diagnostic performance of the angiopoietin levels for sepsis patients. **(A)** Diagnostic performance of angiopoietin-1, **(B)** angiopoietin-2 **(C)** and the angiopoietin-1 to angiopoietin-2 (Ang-1/Ang-2) ratios for sepsis patients. AUC: Area under curve.

**Figure 4.**
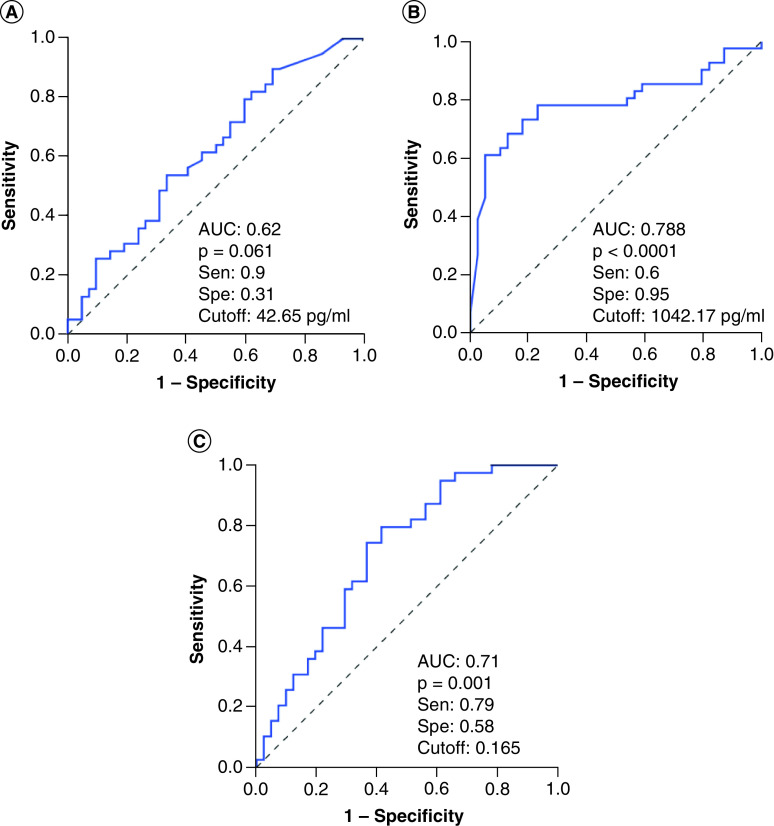
Prognostic performance of the angiopoietin levels for septic shock. **(A)** Prognostic performance of angiopoietin-1, **(B)** angiopoietin-2 **(C)** and the angiopoietin-1 to angiopoietin-2 (Ang-1/Ang-2) ratios in differentiating patients with septic shock from sepsis patients. AUC: Area under curve.

We analyzed the diagnostic performance in differentiating severe sepsis patients from controls individuals at hospital admission, angiopoietin-1 levels and Ang-1/Ang-2 ratios showed a low diagnostic performance (AUC = 0.66), while angiopoietin-2 levels showed a great diagnostic performance (AUC = 0.97) (Figure 3). These results indicated that angiopoietin-2 levels could be an additional potential biomarker for diagnosis of sepsis patients. We also analyzed whether angiopoietin-1 and angiopoietin-2 levels can discriminate patients with septic shock from severe sepsis patients, the results showed that angiopoietin-2 levels could discriminate patients with septic shock from severe sepsis patients (AUC = 0.778) with a cutoff value of 767.3 pg/ml (sensitivity of 78.9% and specificity of 75.6%), while angiopoietin-1 levels and Ang-1/Ang-2 ratios had lower AUC values (AUC = 0.62 and 0.71, respectively) (Figure 4). These results also indicated that angiopoietin-2 levels could be an additional potential biomarker that helps in identifying sepsis patients with higher risk of septic shock development.

## Discussion

Sepsis is a challenge in intensive care due to the complication of host immune response, the change in antibiotic-resistant situations and the emergence of new pathogenic bacteria. Biomarkers for identifying sepsis from other conditions as well as for predicting septic shock in sepsis patients are urgently needed. Angiopoietin has an important role in the pathogenesis of sepsis, involving endothelial cell activation that causes plasma leakage [26,27]. Therefore, angiopoietin-1 and angiopoietin-2 have been considered useful biomarkers to improve early diagnosis, risk stratification and prognosis, especially in the early stages of sepsis progression. In this study, we could show that angiopoietin-2 levels are significantly increased according to the severity of the sepsis development and correlated with important clinical parameters of sepsis such as mean arterial pressure, platelets count, total bilirubin, creatinine, procalcitonin, lactate levels and SOFA score. This study also demonstrated the changes in angiopoietin-1 and -2 levels at hospital admission, 3 and 7 days after hospital admission. In addition, angiopoietin-2 levels also show a good prognostic performance in severe sepsis and in identifying sepsis patients with higher risk of septic shock development.

Angiopoietin-2 has been shown to increase capillary permeability through myosin light chain (MLC) phosphorylation in cultured human microvascular endothelial cells (HMVECs) [19]. Although both angiopoietins bind to the same receptor with equal affinity, angiopoietin-1 and angiopoietin-2 appear to function as an agonist and antagonist pair in most conditions. Our results have confirmed the adverse relationship between the two angiopoietins regarding the levels in different stages of sepsis development and in changing plasma levels during the treatment as well as the correlation with clinical parameters. Previous studies have shown that angiopoietin-1 activates the TIE-2 receptor complex on vascular endothelial cells and appears to promote vascular stability, reduce adhesion molecule expression, and promote endothelial cell survival [27–29], while angiopoietin-2 initially blocks TIE-2 receptors and destabilizes blood vessels, increases adhesion molecule expression and reduces inflammation [28,30]. Therefore, homeostatic signalling through the impairment of TIE-2 phosphorylation contributes to vascular leakage and inflammation. In addition, angiopoietin-2 has been shown to act as a partial TIE-2 agonist and contribute to endothelial cell survival [31], and that is therefore an important mediator in sepsis.

The current study shows that angiopoietin-2 levels were significantly increased in sepsis patients and those with septic shock. This result is in line with a study showing that plasma angiopoietin-2 levels were significantly increased in children with septic shock compared with healthy children and those with critical SIRS and sepsis, and circulating angiopoietin-2 levels were correlated with the severity and complications of the disease [15]. Another study showed that serum angiopoietin-2 levels in patients with systemic inflammatory response, severe infection, sepsis and septic shock were higher than in the control group and were highest in the septic shock group [32]. The study also found that the angiopoietin-1/-2 ratio, angiopoietin-1/TIE-2 ratio and MEDS score were independent predictors of 28-day mortality in patients with sepsis [32]. Similarly, a study investigated serum angiopoietin-2 levels of patients with sepsis and those with severe sepsis and septic shock and showed higher angiopoietin-2 levels in severe sepsis and septic shock, compared with those with sepsis without complications. The higher angiopoietin-2 levels were also positively correlated with the number of organs in failure and were increased during metabolic acidosis and acute coagulopathy [33].

In sepsis, angiopoietin-2 is upregulated and antagonizes angiopoietin-1. In contrast to angiopoietin-2, plasma angiopoietin-1 levels were significantly reduced in children with septic shock compared with those with critical SIRS and sepsis [15]. A previous study showed that lower plasma angiopoietin-1 levels at hospital admission were associated with an increased risk of mortality. Survivors from sepsis had higher angiopoietin-1 levels and lower angiopoietin-2 levels compared with individuals without infection. Plasma angiopoietin-2 levels correlate with clinical signs of organ dysfunction and molecular markers of endothelial cell activation. These results suggest the use of angiopoietins as clinically informative biomarkers of disease severity and patient outcomes in severe sepsis [34].

A Korean study investigated the role of angiopoietin as a biomarker for predicting sepsis by evaluating the association between plasma angiopoietin and various inflammatory cytokines and mortality in patients with severe sepsis. The results showed that plasma angiopoietin-2 levels were correlated with IL-6 and TNF-α. Plasma angiopoietin-2 levels and angiopoietin-2/1 ratio were correlated with the SOFA score and showed a good indicator of 28-day mortality in sepsis patients [35]. In addition, elevated plasma levels of angiopoietin-2 and TIE-2 receptors have been observed in adults with congestive heart failure [17] and in adults with acute sepsis-induced lung injury, and this elevation contributes to endothelial damage in vascular endothelial cells [18]. Plasma angiopoietin-2 levels are also significantly correlated with worsening indices of lung function but recombinant angiopoietin-1 can reverse these effects [18]. Therefore, these data suggest that the angiopoietin-2/TIE-2 receptor complex may be an important target in patients with septic shock and MODS. Angiopoietin-2 can be released from endothelial cells stimulated by inflammatory ligands, such as TNF-α [36]. As such, angiopoietin-2 may serve as an important biomarker of endothelial injury. Similarly, our results also highlight angiopoietin-2 as a potential biomarker for the prognosis of septic shock development. A limitation of the current study was the lack of APACHE-II scores and procalcitonin levels of the control individuals thus the additional analysis to identify the optimal cutoff of angiopoietin-2 for discriminating sepsis has not been performed.

## Conclusion

In conclusion, our data showed that angiopoietin-2 levels were significantly increased while angiopoietin-1 levels were decreased in patients with septic shock compared with those with severe sepsis. Plasma angiopoietin-2 levels and angiopoietin-2/1 ratios were significantly correlated with important clinical parameters such as platelet counts, procalcitonin, lactate levels and indicators of septic shock such as SOFA score. Plasma angiopoietin-2 levels may serve as an additional biomarker for severe sepsis and septic shock.

**Summary points**

- Septic shock is a serious disease with a high mortality rate, ranking first in the intensive care units.
- Angiopoietin-1 and -2 are essential for embryonic and postnatal angiogenesis and stabilization and thus are associated with sepsis-induced multiple organ failure.
- The study investigated the plasma levels of angiopoietin-1 and -2 levels and their association with clinical outcomes of sepsis in plasma from 105 patients with severe sepsis by ELISA.
- Angiopoietin-2 levels were significantly increased in both severe sepsis patients and those with septic shock compared with control individuals (p < 0.0001) and were higher in patients with septic shock compared with severe sepsis patients (p < 0.0001).
- Angiopoietin-2 levels were correlated with mean arterial pressure and platelets counts, total bilirubin levels, creatinine levels, procalcitonin levels, lactate levels and SOFA score.
- Angiopoietin-2 levels accurately discriminated for sepsis with an AUC = 0.97 and septic shock in severe sepsis patients (AUC = 0.778).
- Plasma angiopoietin-2 levels may serve as an additional biomarker for severe sepsis and septic shock.

## References

[1] Vincent JL, Marshall JC, Namendys-Silva SA Assessment of the worldwide burden of critical illness: the intensive care over nations (ICON) audit. Lancet Respir. Med. 2(5), 380–386 (2014). 24740011 10.1016/S2213-2600(14)70061-X

[2] Esposito S, De Simone G, Boccia G, De Caro F, Pagliano P. Sepsis and septic shock: new definitions, new diagnostic and therapeutic approaches. J. Glob. Antimicrob. Resist 10, 204–212 (2017). 28743646 10.1016/j.jgar.2017.06.013

[3] Hotchkiss RS, Moldawer LL, Opal SM, Reinhart K, Turnbull IR, Vincent JL. Sepsis and septic shock. Nat. Rev. Dis. Primers 2, 16045 (2016). 28117397 10.1038/nrdp.2016.45 PMC5538252

[4] Spronk PE, Zandstra DF, Ince C. Bench-to-bedside review: sepsis is a disease of the microcirculation. Crit. Care 8(6), 462–468 (2004). 15566617 10.1186/cc2894 PMC1065042

[5] Lush CW, Kvietys PR. Microvascular dysfunction in sepsis. Microcirculation 7(2), 83–101 (2000). 10802851 10.1038/sj.mn.7300096

[6] Cecconi M, Evans L, Levy M, Rhodes A. Sepsis and septic shock. Lancet 392(10141), 75–87 (2018). 29937192 10.1016/S0140-6736(18)30696-2

[7] Angus DC, van der Poll T. Severe sepsis and septic shock. N. Engl. J. Med. 369(9), 840–851 (2013). 23984731 10.1056/NEJMra1208623

[8] Dolin HH, Papadimos TJ, Chen X, Pan ZK. Characterization of pathogenic sepsis etiologies and patient profiles: a novel approach to triage and treatment. Microbiol. Insights 12, 1178636118825081 (2019). 30728724 10.1177/1178636118825081 PMC6350122

[9] Barichello T, Generoso JS, Singer M, Dal-Pizzol F. Biomarkers for sepsis: more than just fever and leukocytosis-a narrative review. Crit. Care 26(1), 14 (2022). 34991675 10.1186/s13054-021-03862-5 PMC8740483

[10] Sullivan SM, Von Rueden KT. Using Procalcitonin in Septic Shock to Guide Antibacterial Therapy. Dimens. Crit. Care Nurs. 35(2), 66–73 (2016). 26836597 10.1097/DCC.0000000000000164

[11] Fagiani E, Christofori G. Angiopoietins in angiogenesis. Cancer Lett. 328(1), 18–26 (2013). 22922303 10.1016/j.canlet.2012.08.018

[12] Eklund L, Saharinen P. Angiopoietin signaling in the vasculature. Exp. Cell Res. 319(9), 1271–1280 (2013). 23500414 10.1016/j.yexcr.2013.03.011

[13] Fiedler U, Scharpfenecker M, Koidl S The Tie-2 ligand angiopoietin-2 is stored in and rapidly released upon stimulation from endothelial cell Weibel-Palade bodies. Blood 103(11), 4150–4156 (2004). 14976056 10.1182/blood-2003-10-3685

[14] Liu XW MT, Liu W, Cai Q Sustained increase in angiopoietin-2, heparin-binding protein, and procalcitonin is associated with severe sepsis. J. Crit. Care 45, 14–19 (2018). 29413717 10.1016/j.jcrc.2018.01.010

[15] Giuliano JS LP Jr, Harmon K, Wong HR Admission angiopoietin levels in children with septic shock. Shock 28(26), 650–654 (2007). 18092380 PMC2754128

[16] Yu X, Sha J, Xiang S Suppression of KSHV-induced angiopoietin-2 inhibits angiogenesis, infiltration of inflammatory cells, and tumor growth. Cell Cycle 15(15), 2053–2065 (2016). 27294705 10.1080/15384101.2016.1196303 PMC4968962

[17] Chong AY, Caine GJ, Freestone B, Blann AD, Lip GY. Plasma angiopoietin-1, angiopoietin-2, and angiopoietin receptor tie-2 levels in congestive heart failure. J. Am. Coll. Cardiol. 43(3), 423–428 (2004). 15013125 10.1016/j.jacc.2003.08.042

[18] Parikh SM. Dysregulation of the angiopoietin-Tie-2 axis in sepsis and ARDS. Virulence 4(6), 517–524 (2013). 23652985 10.4161/viru.24906 PMC5359737

[19] Parikh SM, Mammoto T, Schultz A Excess circulating angiopoietin-2 may contribute to pulmonary vascular leak in sepsis in humans. PLOS Med 3(3), e46 (2006). 16417407 10.1371/journal.pmed.0030046 PMC1334221

[20] David S, Mukherjee A, Ghosh CC Angiopoietin-2 may contribute to multiple organ dysfunction and death in sepsis*. Crit. Care Med. 40(11), 3034–3041 (2012). 22890252 10.1097/CCM.0b013e31825fdc31 PMC3705559

[21] Singer M, Deutschman CS, Seymour CW The third international consensus definitions for sepsis and septic shock (Sepsis-3). JAMA 315(8), 801–810 (2016). 26903338 10.1001/jama.2016.0287 PMC4968574

[22] Mapalagamage M, Handunnetti SM, Wickremasinghe AR High Levels of Serum Angiopoietin 2 and Angiopoietin 2/1 Ratio at the Critical Stage of Dengue Hemorrhagic Fever in Patients and Association with Clinical and Biochemical Parameters. J. Clin. Microbiol. 58(4), e00436–19 (2020). 31941693 10.1128/JCM.00436-19 PMC7098750

[23] Yu WK, McNeil JB, Wickersham NE, Shaver CM, Bastarache JA, Ware LB. Angiopoietin-2 outperforms other endothelial biomarkers associated with severe acute kidney injury in patients with severe sepsis and respiratory failure. Crit. Care 25(1), 48 (2021). 33541396 10.1186/s13054-021-03474-z PMC7859898

[24] Alrafiah A, Alofi E, Almohaya Y Angiogenesis biomarkers in ischemic stroke patients. J. Inflamm. Res. 14, 4893–4900 (2021). 34588795 10.2147/JIR.S331868 PMC8473716

[25] Knaus WA, Draper EA, Wagner DP, Zimmerman JE. APACHE II: a severity of disease classification system. Crit. Care Med. 13(10), 818–829 (1985). 3928249

[26] Parikh SM. The Angiopoietin-Tie2 Signaling Axis in Systemic Inflammation. J. Am. Soc. Nephrol. 28(7), 1973–1982 (2017). 28465380 10.1681/ASN.2017010069 PMC5491297

[27] Thurston G, Rudge JS, Ioffe E Angiopoietin-1 protects the adult vasculature against plasma leakage. Nat. Med. 6(4), 460–463 (2000). 10742156 10.1038/74725

[28] Maisonpierre PC, Suri C, Jones PF Angiopoietin-2, a natural antagonist for Tie2 that disrupts *in vivo* angiogenesis. Science 277(5322), 55–60 (1997). 9204896 10.1126/science.277.5322.55

[29] Kim I, Moon SO, Park SK, Chae SW, Koh GY. Angiopoietin-1 reduces VEGF-stimulated leukocyte adhesion to endothelial cells by reducing ICAM-1, VCAM-1, and E-selectin expression. Circ. Res. 89(6), 477–479 (2001). 11557733 10.1161/hh1801.097034

[30] Bhandari V, Choo-Wing R, Lee CG Hyperoxia causes angiopoietin 2-mediated acute lung injury and necrotic cell death. Nat. Med. 12(11), 1286–1293 (2006). 17086189 10.1038/nm1494 PMC2768268

[31] Kim I, Kim JH, Moon SO, Kwak HJ, Kim NG, Koh GY. Angiopoietin-2 at high concentration can enhance endothelial cell survival through the phosphatidylinositol 3′-kinase/Akt signal transduction pathway. Oncogene 19(39), 4549–4552 (2000). 11002428 10.1038/sj.onc.1203800

[32] Fang Y, Li C, Shao R, Yu H, Zhang Q, Zhao L. Prognostic significance of the angiopoietin-2/angiopoietin-1 and angiopoietin-1/Tie-2 ratios for early sepsis in an emergency department. Crit. Care 19, 367 (2015). 26463042 10.1186/s13054-015-1075-6 PMC4604731

[33] Lymperopoulou K VD, Kotsaki A, Antypa E Angiopoietin-2 associations with the underlying infection and sepsis severity. 73(71), 163–168 (2015). 10.1016/j.cyto.2015.01.022 25748839

[34] Ricciuto DR dSC, Hawkes M, Toltl LJ Angiopoietin-1 and angiopoietin-2 as clinically informative prognostic biomarkers of morbidity and mortality in severe sepsis. Crit. Care Med. 39(4), 702–710 (2011). 21242795 10.1097/CCM.0b013e318206d285

[35] Seol CH, Yong SH, Shin JH The ratio of plasma angiopoietin-2 to angiopoietin-1 as a prognostic biomarker in patients with sepsis. Cytokine 129, 155029 (2020). 32059166 10.1016/j.cyto.2020.155029

[36] Fiedler U, Reiss Y, Scharpfenecker M Angiopoietin-2 sensitizes endothelial cells to TNF-alpha and has a crucial role in the induction of inflammation. Nat. Med. 12(2), 235–239 (2006). 16462802 10.1038/nm1351

