# Supplementary material for: Prognostic significance of the angiopoietin-2 for early prediction of septic shock in severe sepsis patients
Source: Future Sci OA. 2023 Jan 27;8(10):FSO825. doi: 10.2144/fsoa-2022-0077 (PMC9979161; doi:10.2144/fsoa-2022-0077)
Supplement: Supplementary file 1 [file fsoa-08-825-s1.docx]

**Supplementary Table 1: Correlation of Angiopoietin-1 and Angiopoietin-2 levels with clinical parameters**

| **Clinical parameters** | **Correlation** | **Hospital admission** | | | **3 days after Hospital admission** | | | **7 days after Hospital admission** | | |
| --- | --- | --- | --- | --- | --- | --- | --- | --- | --- | --- |
|  |  | **Angiopoietin-1 levels (pg/ml)** | **Angiopoietin-2 levels (pg/ml)** | **Ang-1/2 Ratio** | **Angiopoietin-1 levels (pg/ml)** | **Angiopoietin-2 levels (pg/ml)** | **Ang-1/2 Ratio** | **Angiopoietin-1 levels (pg/ml)** | **Angiopoietin-2 levels (pg/ml)** | **Ang-1/2 Ratio** |
| Mean Arterial Pressure (mmHg) | ρ (rho) | 0.13 | **-0.42** | **0.28** | 0.13 | **-0.3** | **0.23** | **0.49** | -0.15 | **0.43** |
|  | P value | 0.24 | **<0.0001** | **0.012** | 0.28 | **0.012** | **0.05** | **0.029** | 0.52 | **0.056** |
| White blood cells | ρ (rho) | 0.07 | 0.08 | 0.06 | 0 | 0.19 | -0.07 | 0.17 | 0.06 | 0.17 |
|  | P value | 0.54 | 0.51 | 0.63 | 1 | 0.18 | 0.63 | 0.28 | 0.7 | 0.31 |
| Platelets (G/L) | ρ (rho) | **0.29** | **-0.232** | **0.36** | **0.62** | **-0.57** | **0.69** | **0.43** | -0.08 | 0.25 |
|  | P value | **0.012** | **0.047** | **0.0014** | **<0.0001** | **<0.0001** | **<0.0001** | **0.006** | 0.63 | 0.14 |
| Hematocrit (%) | ρ (rho) | -0.08 | 0.04 | -0.11 | 0.1 | -0.02 | 0.1 | -0.12 | **-0.37** | 0.036 |
|  | P value | 0.47 | 0.73 | 0.34 | 0.48 | 0.89 | 0.48 | 0.45 | **0.023** | 0.83 |
| Total Bilirubin (µmol/L) | ρ (rho) | **-0.38** | **0.37** | **-0.49** | **-0.34** | **0.51** | **-0.47** | **-0.388** | **0.33** | **-0.46** |
|  | P value | **0.0017** | **0.0025** | **<0.0001** | **0.02** | **0.0003** | **0.0012** | **0.03** | **0.059** | **0.0066** |
| Blood Creatinine (µmol/L) | ρ (rho) | -0.05 | **0.43** | -0.23 | **-0.31** | **0.33** | **-0.34** | -0.22 | **0.45** | **-0.37** |
|  | P value | 0.67 | **0.00012** | 0.06 | **0.018** | **0.011** | **0.008** | 0.15 | **0.003** | **0.015** |
| Procalcitonin (ng/ml) | ρ (rho) | -0.096 | **0.56** | **-0.29** | **-0.28** | **0.42** | **-0.38** | -0.04 | **0.39** | -0.18 |
|  | P value | 0.39 | **<0.0001** | **0.01** | **0.03** | **0.0004** | **0.0016** | 0.8 | **0.008** | 0.25 |
| Albumin (g/L) | ρ (rho) | 0.07 | -0.15 | 0.104 | 0.04 | -0.18 | 0.12 | -0.1 | **-0.36** | 0.07 |
|  | P value | 0.53 | 0.18 | 0.36 | 0.76 | 0.15 | 0.35 | 0.55 | **0.014** | 0.63 |
| Lactate (mmol/L) | ρ (rho) | -0.16 | **0.47** | **-0.29** | **-0.49** | **0.46** | **-0.56** | -0.13 | -0.17 | 0.06 |
|  | P value | 0.25 | **0.0004** | **0.037** | **0.0013** | **0.0024** | **0.00016** | 0.65 | 0.54 | 0.82 |
| SOFA score | ρ (rho) | -0.04 | **0.41** | -0.18 | -0.07 | -0.01 | 0.013 | 0.09 | 0.18 | -0.09 |
|  | P value | 0.7 | **0.0002** | 0.11 | 0.58 | 0.93 | 0.9 | 0.54 | 0.2 | 0.52 |
| APACHE II score | ρ (rho) | 0.08 | 0.05 | 0.08 | 0.09 | 0.13 | 0.05 | -0.02 | 0.23 | -0.07 |
|  | P value | 0.48 | 0.65 | 0.49 | 0.47 | 0.29 | 0.66 | 0.86 | 0.09 | 0.6 |

The correlations between the pair of studied parameters were calculated by using Spearman's rank correlation coefficient. Spearman's rho (ρ) and *P* values are presented.
